# Conservative treatment of left ventricular pseudoaneurysm after mitral valve replacement due to early left ventricular rupture: a case report

**DOI:** 10.1186/s13019-021-01436-8

**Published:** 2021-04-06

**Authors:** Qun-Jun Duan, Cui-Ting Duan, Wei-Jun Yang, Ai-Qiang Dong

**Affiliations:** 1 Department of Cardiovascular Surgery, School of Medicine, Second Affiliated Hospital of Zhejiang University, #88 Jiefang Road, Hangzhou, 310009 China; 2 Department of Nursing, Hangzhou Medical College, Hangzhou, China

**Keywords:** Left ventricular pseudoaneurysm, Mitral valve replacement, Left ventricle rupture

## Abstract

**Background:**

Left ventricular pseudoaneurysm due to early left ventricle rupture is a serious complication after cardiac surgery. Urgent surgery is recommended in most cases with a high mortality rate. Conservative treatment of a left ventricular pseudoaneurysm due to early left ventricle rupture is very rare.

**Case presentation:**

We present a 61-year-old woman with left ventricular pseudoaneurysm after mitral valve replacement due to early left ventricle rupture. This patient was treated in a conservative approach. This patient had an uneventful recovery. She was in good condition and remained asymptomatic 3.5 years after mitral valve surgery.

**Conclusion:**

This case suggests that medical treatment left ventricular pseudoaneurysm patients has a limited but acceptable role in selected and unusual circumstances.

**Supplementary Information:**

The online version contains supplementary material available at 10.1186/s13019-021-01436-8.

## Background

Left ventricular pseudoaneurysm (LVPA) is a contained left ventricle (LV) rupture which is encircled by adherent pericardium or scar tissue. It is a potentially fatal condition with a high risk of rupture. As a rare and serious complication after cardiac surgery, LVPA mainly occurs after mitral valve surgery. Urgent surgical intervention is recommended as the first choice in most cases with a high mortality rate of 57.4% [1]. We report a case with an LVPA after mitral valve replacement (MVR) due to early LV rupture. This patient was successfully treated in a conservative approach.

## Case presentation

A 61-year-oldwoman with left hemiparalysis and seizure caused by previous cerebral infarction was referred with fatigue and progressive dyspnoea on exertion. She had severe cardiogenic dyscrasia with a body weight of 32 kg and body mass index of 12.8 kg/m^2^. She was admitted for MVR due to severe combined mitral valve stenosis and insufficiency. Preoperative transthoracic echocardiography (TTE) revealed rheumatic chordal shortening and severe mitral leaflet thickening with a preserved LV function of 59%. Electrocardiogram showed persistent atrial fibrillation. At the time of surgery through a median sternotomy, the valve was assessed unsuitable for repair. The anterior mitral leaflet was totally removed. Most of the posterior mitral leaflet was preserved. An MVR was performed with a 27 mm Mosaic® bioprosthesis (Medtronic, Minneapolis, MN, USA). The valve was implanted using everted interrupted pledgeted polyester sutures. After discontinuation of cardiopulmonary bypass and closure of the sternum, the drapes were removed. However, before the patient was transferred out of operating room, a total of 700 ml bleeding was observed from the pericardial drainage tube during a short period of 20 min. The sternum was immediately re-opened. Careful exploration revealed no major bleeding from either the mediastinum or the pericardial cavity. After 30 min of observation with no major bleeding, the sternum was closed. Post-operative bleeding in the subsequent 24 h from the pericardial drainage tube was 200 ml. She was extubated the second day after surgery. The postoperative course was uneventful.

**Fig. 1:**
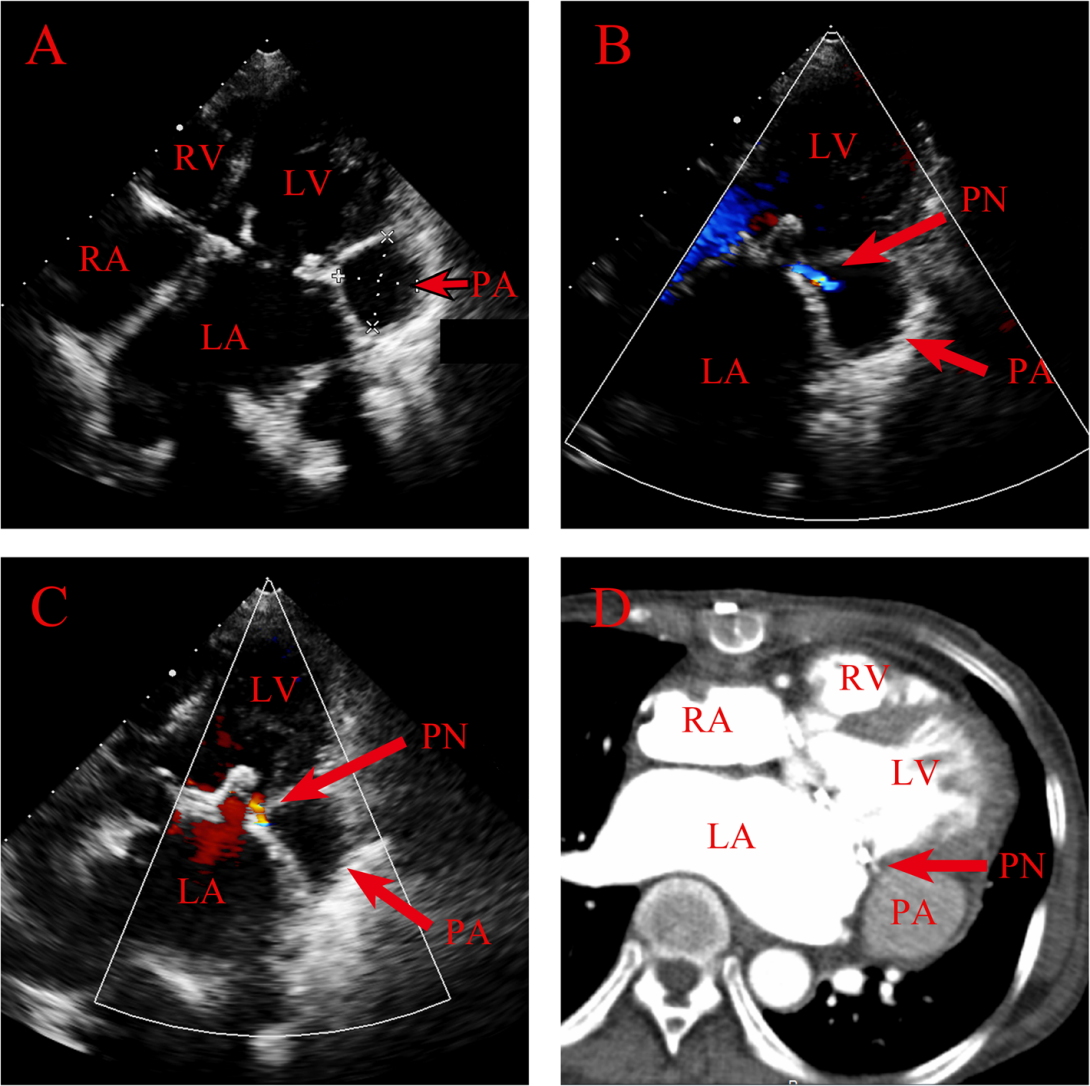
**a** Two-dimensional transthoracic echocardiogram in apical four-chamber view showed an aneurysm-like structure measuring (29 × 25 mm) suggestive of a submitral aneurysm. Color Doppler imaging revealed blood inflow from LV to pseudoaneurysm (**b**) and from pseudoaneurysm to LV (**c**) through a narrow neck (measuring 3 mm) in systole. **d** Contrast-enhanced cardiac computed tomography confirmed the pseudoaneurysm. RV: right ventricle. RA: right atrium. LV: left ventricle. LA: left atrium. PA: pseudoaneurysm. PN: pseudoaneurysm neck


However, we noted an aneurysm-like cavity (29 × 25 mm) proximal to mitral annulus on the posteroleteral wall of the LV on postoperative day 10 during routine postoperative TTE (Fig. 1a). Color Doppler imaging revealed blood flow communication between LVPA and LV through a narrow neck (measuring 3 mm), which had inflow synchronizing a cardiac cycle, from LV to cavity in systole (Fig. 1b, Video 1) and from cavity to LV in diastole (Fig. 1c, Video 1). Left ventricular pseudoaneurysm after LV rupture was diagnosed. In consideration of massive bleeding in the operating room and uneventful recovery after surgery, LV rupture was believed to take place in the operating room. Contrast-enhanced cardiac computed tomography confirmed a pseudoaneurysm arising from the posteroleteral wall of the LV through a narrow neck (Fig. 1d).

Additional file 1: **Video 1.** Color Doppler imaging revealed blood flow communication between left ventricle pseudoaneurysm and left ventricle through a narrow neck (measuring 3 mm), which had inflow synchronizing a cardiac cycle, from left ventricle to pseudoaneurysm in systole and from pseudoaneurysm to left ventricle in diastole.

**Fig. 2:**
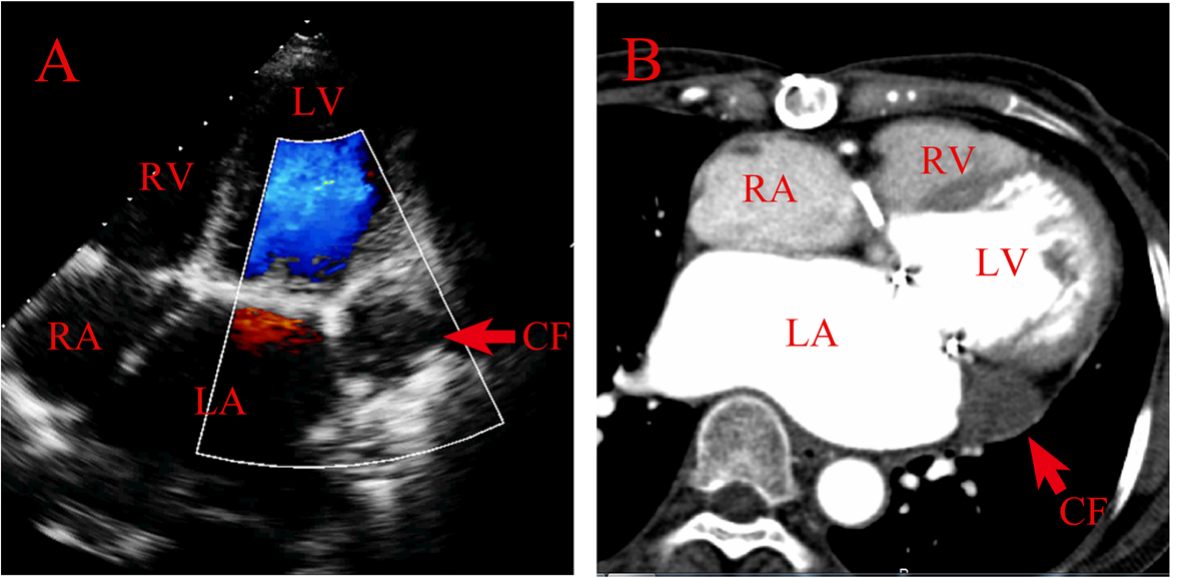
**a** Follow-up transthoracic echocardiogram in apical four-chamber view showed no blood communication between the clot-filled pseudoaneurysm and the left ventricle. The psedoanurysmal sac was filled with clot. **b** Follow-up enhanced computed tomography revealed no enhancement of the pseudoaneurysm. RV: right ventricle. RA: right atrium. LV: left ventricle. LA: left atrium. CF: clot formation


Based on these findings, surgery was recommended immediately. However, both the patient and her family rejected our proposal due to high surgical mortality and her worsened frailty. Hence, we started the optimal conservative management. She was started on warfarin to prolong the prothrombin time (international normalized ratio) to 2.0. Her blood pressure was monitored and controlled. She was discharged on postoperative day 15 with stable clinical condition under the administration of warfarin because of persistent atrial fibrillation. Six months after MVR, TTE showed no blood communication between the clot-filled pseudoaneurysm and the LV (Fig. 2a, Video 2). Enhanced computed tomography (Fig. 2b) revealed no enhancement of the pseudoaneurysm. Thrombus formation in the pseudoaneurysm was confirmed. The size of the pseudoaneurysm decreased to 20 × 22 mm. At present, 4 years after surgery, she remained asymptomatic with regular warfarin therapy.

Additional file 2: **Video 2.** Follow-up transthoracic echocardiogram in apical four-chamber view showed no blood communication between the clot-filled pseudoaneurysm and the left ventricle.

## Discussion and conclusion

Left ventricular pseudoaneurysm after mitral valve surgery is a type of rare and lethal condition derived from rupture of LV. It can take place after both mitral valve repair [1] and replacement. The incidence of this complication was 0.56% and the mortality rate was 57.4% [1]. Reoperation, endocarditis, severe annular calcification, poor visualization of the operating field, low-sized LV and oversizing of prosthetic valves are known to be risk factors. Other causes of rupture of the LV free wall include the untethering of the fibrous structures of the LV during resection of mitral leaflets, an increase in LV contractility after aortic crossclamping, enhanced LV wall stress with the support of inotropic agents, and other mechanical trauma between the free wall and the papillary muscles, such as rubber catheter wedging or metal pump suction during valve replacement [2].

Left ventricular pseudoaneurysm develops only when LV rupture is barely contained by adherent pericardium or scar tissue. Left ventricular rupture was classified according to the timing of rupture [3]. Early rupture is defined as occurring in the operating room any time after discontinuation of cardiopulmonary bypass. Delayed rupture manifests in the recovery room usually hours to days postoperatively. Late rupture occurs days to years after the valve replacement and presents as pseudoaneurysm. In consideration of massive bleeding in the operating room and uneventful recovery after surgery in this case, LV rupture was believed to take place in the operating room. Therefore, this case had an early LV rupture which was encapsulated by an LVPA. The early rupture comprises the majority of LV ruptures following MVR. The mortality rate in these patients is extremely high despite early treatment. The majority of LVPA cases that were reported in the literature presented as a late complication after MVR [4–6]. This case is exceptionally rare because LVPA developed as a kind of early LV rupture.

Although prompt surgical repair have been recommended as the method of first choice to prevent LVPA rupture, surgical repair may be hesitant because of the high mortality rate. In consideration of her extremely low body mass index and advanced comorbidity in our case, immediate surgery of this LVPA was believed to have an exceedingly high mortality rate. However, the aneurysmal neck and dimension of the LVPA was fortunately small, which made a conservative management feasible and successful despite continuation of warfarin therapy. During the long-term follow-up, the patient showed stable clinical and hemodynamic conditions. This interesting case suggests that conservative treatment can be an alternative in appropriate patients.

Although 30–40% of untreated pseudoaneurysms rupture in the first year [7], the treatment strategy remains controversial [1, 8–10]. Surgery is suggested for patients with large or expanding false aneurysms. Conservative strategies yielded positive results in several cases [1, 8–10]. Sakai et al. reported that 7 out of 8 patients with a LVPA after mitral valve repair were managed conservatively, and indicated that conservative management may be possible if the pseudoaneurysm is small and its neck is very narrow [9]. Factors that favor a conservative management include high surgical risks, no clinical symptom, small pseudoaneurysm less than 3 cm, small aneurysmal neck and stable echocardiographic and clinical manifestations [10, 11]. Percutaneous approaches to closure of LVPA have recently been described [12, 13]. For a population of elderly, fragile patients with severe comorbidities, percutaneous closure of the LVPA seems to be a viable minimally invasive option. Anatomy, the size and the width of the neck of the LVPA should be considered when an optimal approach is planned. The disadvantage of these devices is the possibility of mechanical compression of adjacent epicardial coronary vessels.

We presented a rare case of conservatively managed LVPA after early LV rupture. This case suggests that medical treatment LVPA patients has a limited but acceptable role in selected and unusual circumstances.

## Data Availability

Not applicable.

## Abbreviations

LVPA: Left ventricular pseudoaneurysm
RV: Right ventricle
RA: Right atrium
LV: Left ventricle
LA: Left atrium
PA: Pseudoaneurysm
PN: Pseudoaneurysm neck
MVR: Mitral valve replacement
TTE: Transthoracic echocardiography
